# Exploring Edible Insects: From Sustainable Nutrition to Pasta and Noodle Applications—A Critical Review

**DOI:** 10.3390/foods13223587

**Published:** 2024-11-10

**Authors:** Carlos Gabriel Arp, Gabriella Pasini

**Affiliations:** 1 Centro de Investigación y Desarrollo en Criotecnología de Alimentos (CIDCA), Facultad de Ciencias Exactas-Universidad Nacional de La Plata, Comisión de Investigaciones Científicas de la Provincia de Buenos Aires, Consejo Nacional de Investigaciones Científicas y Técnicas, 47 y 116, 1900 La Plata, Buenos Aires, Argentina; 2 Department of Agronomy, Food, Natural Resources, Animals and the Environment, University of Padova, Viale Dell’Università 16, 35020 Legnaro, Padova, Italy; gabriella.pasini@unipd.it

**Keywords:** novel foods, entomophagy, insect-based pasta, insect-based noodles, bioactive

## Abstract

Edible insects provide an alternative source of high-quality proteins, essential lipids, minerals, and vitamins. However, they lack the acceptability and consumption rates of more common staple foods. In contrast, pasta and noodles are globally appreciated foods that are consumed across various cultures. These products contribute greatly to the population’s energy intake but generally lack essential nutrients. Recently, edible insects have gained in popularity due to their numerous benefits, both environmental and nutritional. Current research indicates that incorporating edible insect ingredients into pasta and noodle formulations enhances their nutritional quality by increasing protein and fiber content and reducing carbohydrates. However, adding new ingredients to enrich common foods often carries technological and sensory challenges, such as changes in processing parameters, texture, flavor, and appearance. Technology assessment, scientific research, information campaigns, and public policies can help overcome these issues. This review aims to summarize the benefits of entomophagy (the consumption of insects as food) for sustainability, nutrition, and health; highlight the potential of pasta and noodles as carriers of nutritious and bioactive ingredients, including insects; and critically address the advancements in insect-enriched pasta and noodle technology, identifying current challenges, knowledge gaps, and opportunities.

## 1. Introduction

In recent years, edible insects have become an emerging trend in the search for greener and more environmentally friendly foods. They constitute alternative, more sustainable sources of nutrients than those coming from traditional agriculture and cattle practices. Insects have been used as food for millennia. They are rich in several nutrients, particularly in high-quality proteins, essential lipids, minerals, and vitamins. In this way, the practice of consuming insects (known as entomophagy) complements the diets of millions of people around the world. At the same time, entomophagy is considered a disgusting or repellent activity for certain populations. This is particularly true in countries or regions where it is unfamiliar [1] or where it was historically replaced by other food supplies (such as cattle and agriculture) for a long time [2]. In this context, insects were mostly eaten whole, seasoned, and/or cooked in diverse forms, and it was only in the last few years that processing technology paid more attention to them.

On the other hand, cereal products such as pasta and noodles are well-established staple foods, appreciated and produced all around the globe. They have been prepared using traditional artisanal procedures for thousands of years, accompanied by a long history of technological development for their industrial processing. Nevertheless, the consumption of these products, especially those made from refined flour, contributes mainly to energy intake. For this reason, these products often need to be complemented with other meals and nutrient sources (especially proteins) to maintain a healthy diet [3]. This is particularly important in gluten-free pasta and noodles, where the nutritional quality is often poorer in comparison with their gluten-containing counterparts [4,5,6].

**Figure 1:**
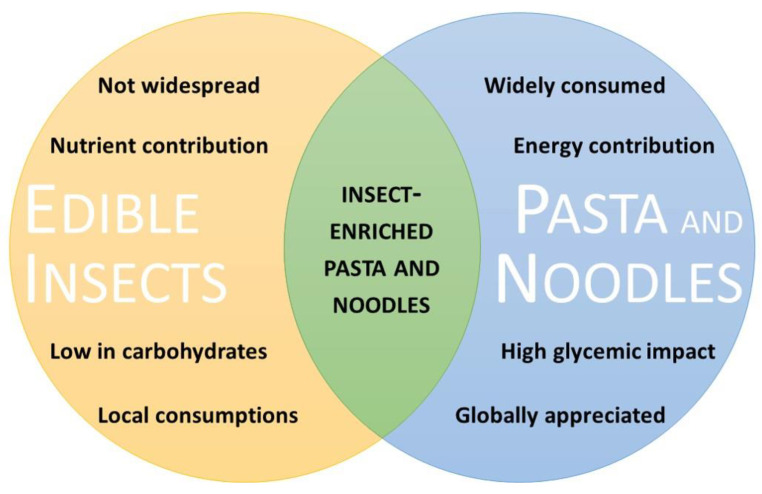
Complementary aspects of edible insects and pasta and noodles.

Considering the current trends and the complementary situations of these food products, several authors have started to study the possibility of producing insect-enriched pasta and noodles (Figure 1). The first part of this review aims to summarize insects’ contributions towards achieving sustainability, nutrition, and health and to highlight the potential of cereal-based products, especially pasta and noodles, as carriers of nutritious and bioactive ingredients. Then, the review critically addresses the most recent advances in insect-enriched pasta and noodles (both gluten and gluten-free), identifying current challenges, knowledge gaps, and stimulating opportunities.

## 2. Insects as Food

### 2.1. A Way Towards Sustainability

Using insects as food ingredients is a trend that is rising quickly, together with other alternative protein sources such as plant, fungi, and algae proteins, as well as technologies such as cultured meat and 3D printing [7,8,9]. Several countries and regions have already settled the foundations of their regulations for insect production, especially in the EU, Asia, and North America, and many others in Africa and South America are currently on their way to doing so [10,11].

Insects have been used as a source of nutrients for millennia. Ancient populations have benefited from several advantages, possibly since their origins [12,13,14]. However, with the emergence of new ways to obtain food, such as the rise of agriculture, the insects were moved from a source of nutrients to a plague that damaged crops and then largely obliterated [2]. Nevertheless, they were never completely forgotten. Several cultures have preserved the ancient tradition of entomophagy all around the world, and this practice is about to play a key role in modern societies [9,15].

Edible insect species are present everywhere. They can be collected in the wild or reared in controlled conditions, such as in small facilities or in massive farms. When reared, room temperature, relative humidity, and even light:dark ratio are variables that can be standardized to optimize productivity (mass yield, survival rate, reproduction, and so on). However, not only do different insect species require distinct optimal conditions, but also the different stages of their development do, i.e., growth, mating, oviposition, egg hatching, etc. In a very interesting review, Morales-Ramos et al. [16] provide a detailed summary of these different conditions for four commonly reared species of edible insects (*Acheta domesticus*, *Tenebrio molitor*, *Hermetia illucens*, and *Musca domestica*). Nevertheless, more research on this topic is still needed to include more insect species and production schemes to prevent insect disease that could harm production yields and guarantee food safety.

Regarding feed, insects can be fed with organic sources, formulated feed, or suitable by-products from the agri-food industry. The omnivorous nature of many edible insects (e.g., crickets, fly larvae) and their high feed-to-biomass conversion rate make them especially suitable for integration into circular economy schemes. Moreover, their wastes (also known as frass) are mostly dry and easily compostable [2].

**Figure 2:**
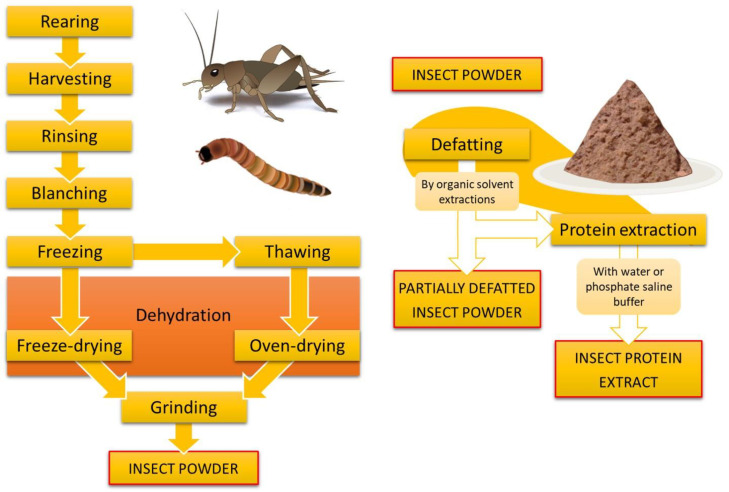
General procedures for the obtention of insect ingredients.

Insects can be used as human food or animal feed. They can be consumed as a whole, with minimal processing (chopped, cooked, seasoned), or transformed into entirely new ingredients (Figure 2) for formulating enriched products. Such versatility regarding easy access and sustainability is comparable only to the vegetal and the fungi but not to the modern cattle industry [2]. Now that the current food chains are being targeted as one of the most responsible for the climate crisis, the ancient practice of consuming insects is rising again as a new opportunity for sustainability. In comparison to cattle, insects have a significantly lower carbon footprint, require less land and resources to be reared (including feed, water, and energy), and are more efficient for reproducing and converting feed into biomass, i.e., food mass [9].

In fact, as it is said in almost every scientific article that focuses on insects as food and feed, one of the main advantages of moving from a traditional cattle industry-based scheme of production to an entomophagy-based one is, precisely, sustainability.

Despite this running trend, there is still a cultural and social threshold to overcome to achieve worldwide acceptance of insect-based foods. Here is where science and technology, in tandem with all parts of the food environment (including authorities, retailers, informative campaigns, funding programs, and even educative systems), can make a substantial difference. Especially by developing new appetizing products, making them accessible, finding more benefits in favor of their consumption, and communicating them in an efficient and clear way [17,18].

### 2.2. A Way Towards Nutrition

Several authors have already established the nutritional properties of edible insects. Making another compilation of studies addressing this issue would be pointless for the aims of this review since there are already plenty of scientific articles and reviews on the nutrient composition of an immensely wide range of species. Pioneer works such as that from Finke [19], one of the first assessing the nutritional composition of several insect species, and the literature research from Rumpold and Schlüter [20], one of the most complete species-based compilation of nutritional compositions so far, have greatly helped the design of new studies and the proliferation of the scientific literature in this regard. However, since the former is focused on the nutritional properties of insects for insectivore animals, and the latter compiles a wide variety of works from diverse fields (entomophagy, entomology, insects as feed), an update to these foundational works focused on insects intended for human consumption should be taken into account. Moreover, since the publication of these two articles, most studies on entomophagy have focused only on a dozen of trending species, i.e., *Gryllus bimaculatus*, *A. domesticus*, *Schistocerca gregaria*, *Locusta migratoria*, *Gryllodes sigillatus*, *Gryllus assimilis* (Orthoptera), *T. molitor*, *Rhynchophorus phoenicis*, *Alphitobius diaperinus*, *Zophobas morio* (Coleoptera), *Bombyx mori*, *Imbrasia oyemensis* (Lepidoptera), *H. illucens*, *M. domestica* (Diptera), *Apis mellifera* (Hymenoptera) [18,21,22,23]. Such studies have led to a range of nutritional features that can be partially generalized into ranges, e.g., 35–60% protein (crude), 13–33% lipids, 4–20% carbohydrates, 5–13% dietary fiber (chitin), and 3–10% ashes (dry basis). Thus, insects would greatly contribute to complementing current diets with essential amino acids [8,24,25,26,27,28], minerals and vitamins [27,28,29,30,31,32], omega-9 fatty acids [31,32,33,34,35,36], and dietary fiber [28,37,38].

Nevertheless, there are still some issues that need to be addressed in order to accurately evaluate the nutritional composition of these ingredients. Insects as food are mostly appreciated because of their protein contents, but most works fail to report accurate values for them [39]. This could be due to many reasons, but mainly to the lack of consensus on how to report this parameter (i.e., crude protein vs protein), the lack of familiarity of the food industry with assessing chitin content on a daily basis (uncommon enzymes and procedures), and the lack of consensus on nitrogen-to-protein conversion factors. Some efforts to address these issues are starting to appear in the literature. Toribio et al. [28] proposed a combination of three methods commonly used in the food industry to characterize cricket powder from *G. assimilis*. The authors stated that evaluating total nitrogen content, nitrogen content in the total dietary fiber mass, and amino acid profiles allows for a quick estimation of nitrogen-to-protein conversion factors, protein content, chitin content, and protein digestibility in a more accurate way. Moreover, even if amino acid profiles cannot be measured on-site, the combination of total nitrogen content and nitrogen content in total dietary fiber assessments would allow for the estimation of the content of available nitrogen from proteins.

However, a consensus on protein evaluation must be seen as a general aim in the field of entomophagy at this initial stage of research. Consequently, more studies focused on obtaining nitrogen-to-protein conversion factors and developing validated methods for routine chitin determination are yet to be addressed, at least in principle, for the species that are currently considered the most suitable for rearing, production, and consumption.

### 2.3. A Way Towards Health

The above-mentioned general nutritional profile, rich in essential amino acids, fatty acids, minerals, and vitamins, is also accompanied by a vast range of biological activities. Just in 2024, several studies confirmed the bioactive potentiality of diverse insect-based products in vivo, in vitro, and in silico, e.g., antioxidant [40,41], antihypertensive [41,42], anti-inflammatory [40,43], antimicrobial [44], antidiabetic [45], antiosteoclastogenic [46], and so on. These new findings are summarized in Table 1. Such features are mostly attributed to their protein hydrolysates and peptides, unsaturated and essential fatty acids, chitin, and even several compounds such as terpenoids, steroids, phenolic compounds, carotenoids, and glycosides; some endogenous and some obtained through diet [47,48,49].

Insights about the bioactive properties of insect-based ingredients have been growing fast in these last few years. This scenario opens the possibility for formulating novel functional foods based on these ingredients, strengthening the motivation for insect-enriched novel foods. For this to be possible, gaining insight into how the bioactive properties of insect ingredients are transferred into food products is crucial. Thus, further research must be conducted to investigate these bioactive properties in the forthcoming developments.

## 3. Pasta and Noodles as Carrier Foods

When it comes to finding a way to convey new beneficial ingredients, enriching staple food products can be a good starting point. Thus, cereal-based products, such as pasta and noodles made from wheat, rice, and maize flour, have been largely studied to formulate new functional products [50]. Cereal-based products are commonly produced using refined cereal flours such as wheat, rice, and corn flours, as well as their isolated starches. In these refined forms, cereals contribute high amounts of easily digestible carbohydrates (starch), comprising from 70 to more than 80% of their dry weight [51,52,53,54] and low amounts of beneficial components such as dietary fiber, vitamins, phenolic compounds, flavonoids, etc. [50].

The difference between pasta and noodles relies mainly on their raw materials. Pasta is typically produced using durum wheat (*Triticum durum*) semolina, the product of a coarse milling of durum wheat [55]. Although their consumption is currently widespread, pasta products are generally associated with the Mediterranean region. Conversely, noodles are generally made from common wheat flour (*Triticum aestivum*) and are associated with the Asian culinary culture [56]. Durum wheat semolina has a coarser particle size, is higher in proteins, and is richer in carotenoids in comparison to common wheat flour. For these reasons, pasta products are firm and dense, require a longer time to cook, and often present an attractive yellow hue [55,57]. Noodles made from common wheat flour are, conversely, softer, short-cooked, and whiter. Both pasta and noodles can include other additional ingredients such as different flours, eggs, and vegetables. They can also be elaborated on using different processing methods, such as laminating and shaping or extrusion. Durum wheat semolina dough exhibits stronger tenacity, so both laminating and shaping, as well as extrusion procedures, are suitable. Common wheat flour dough is softer, so it is generally handled by laminating and shaping. Additionally, both kinds of products can be commercialized fresh or further processed to obtain dry products with a longer shelf life [55,56]. Pasta can also be filled with different ingredients (filled or stuffed pasta) and is often consumed accompanied by vegetable and dairy sauces or as an ingredient for salads. Noodles are typically consumed as spicy soups, stir-fried dishes, or even as sweet desserts and can also be filled with different ingredients (dumplings) [58].

Some authors have reported the effect of the food matrix on the digestion of cereal-based products [59,60,61,62]. Pasta and noodles present a compact and dense matrix produced by kneading, laminating, and/or extruding cereal flours in a relatively restricted water environment. In these conditions, the cohesiveness of the product, i.e., its integrity, highly depends on the development of an elastic, hydrated matrix, in which the partially gelatinized starch granules are mostly entrapped by a continuous gel-like arrangement of gluten proteins [59,62]. Given these characteristics, pasta and noodles are more likely to be used as carriers of different kinds of beneficial ingredients such as antioxidants, proteins, vitamins, soluble dietary fiber, and other enriching ingredients that are less likely to hinder the continuity of the matrix and then affect its integrity [50,63,64,65,66,67]. Moreover, the resulting compact matrix of pasta and noodles is more difficult to disintegrate into small particles in comparison with softer matrices, producing denser bolus and chyme, limiting the diffusion of enzymes and thus slowing carbohydrate digestion [59,68]. For this reason, pasta and noodles are generally more moderate than other cereal-based products when it comes to assessing the implications of their glycemic response. However, the glycemic load and impact of these products are still high. Therefore, they should be consumed in moderation, especially for low-calorie regimens such as those intended for managing non-transmissible chronic pathologies, i.e., type 2 diabetes mellitus or metabolic syndrome [69,70]. Moreover, all cereal-based products are known for being lower in some important nutrients, such as essential amino acids (especially lysin), and their nutritional benefits rely mainly (but not only) on energy intake [71]. Thus, adding new ingredients that can enhance the nutritional profile of these kinds of foods is still a strategic way to develop healthier products.

## 4. Insect-Enriched Pasta and Noodles

### 4.1. A Strategy for Global Nutrition

As stated in the previous sections, pasta and noodle products are present all around the world, as are edible insects. The ubiquity of both products makes their crossover a feasible possibility in theoretical terms. Their combined features can benefit from each other for rendering insect-enriched, good-quality pasta and noodles, whether gluten-free or not.

Some authors have already reported on how the nutritional composition of pasta and noodles is enhanced when incorporating insect products. This includes whole insect powders or their defatted and extracted derivatives (Figure 2). However, given the limited number of studies addressing this issue in the scientific literature, especially those regarding gluten-free products, it is premature to draw general conclusions. This is particularly true because many of these studies were conducted under varying conditions, including differences in terms of pasta ingredients, insect species, insect products, and the level of usage. Additionally, the design of the formulations often lacks the critical details necessary for proper analysis. The basis on which the insect ingredients are added (i.e., insect: flour ratio or insect:full formulation ratio) is often not clear. The same applies to the manner in which the results are presented (i.e., dry basis, wet basis, crude protein or actual protein, conversion factors, dietary fiber, etc.). Nonetheless, some general trends can be observed regarding the effects of incorporating these ingredients, particularly in terms of proteins, fats, and micronutrients.

#### 4.1.1. Wheat-Based Pasta

Duda et al. [72] reported the nutritional value of semolina pasta made with different levels of cricket (*A. domesticus*) powder. In this case, the authors stated that the final pasta formulations contained 5, 10, and 15% addition of cricket powder, although it is not clear if the water was taken into account for those reported levels. However, the authors found significant and progressive increases in the pasta protein (up to 70%), fat, and mineral contents, accompanied by a 13% reduction in carbohydrate content. Although samples with cricket flour had higher energy values, it is worth noting that such contribution shifted from carbohydrates to fats, which could represent a benefit for diets where carbohydrates need to be limited. Çabuk and Yılmaz [73] replaced 15% wheat flour with flour from two different insect species: *L. migratoria* and *T. molitor*, to produce erişte, a traditional Turkish egg pasta. The authors reported 51 and 42% more protein for *L. migratoria* and *T. molitor* pasta, respectively. Moreover, the starch content decreased up to 14% in both cases, and the crude fiber content increased by 175% for the *L. migratoria* pasta and 113% for the *T. molitor* pasta. However, there is no information regarding the fat contents.

On the other hand, Piazza et al. [74] and Hidalgo et al. [75] formulated semolina pasta with *B. mori* powder and *B. mori* protein extracts. When replacing 10% of a mixture of semolina and wheat flour with defatted *B. mori* flour, Piazza et al. [74] found significant increments in the protein (41%), fats (20%), and ash (76%) contents, although in this case the authors did not report values of carbohydrates or dietary fiber. In the same study, similar results were found when semolina and wheat flour were replaced with *B. mori* protein extracts instead of the defatted flour. In this case, the proteins increased by 37%, and fats were similar to the wheat control. Hidalgo et al. [75] found that replacing semolina with 15% salt-soluble proteins from *B. mori* led to 33% more protein, 14% fewer lipids, 14% fewer carbohydrates, and similar dietary fiber values to the semolina control.

In the same study, Hidalgo et al. [75] replaced 15% semolina with *H. illucens* salt-soluble proteins, resulting in pasta with 20% more protein. As for the other components (fats, carbohydrates, and dietary fiber), the results were similar to those found with *B. mori* extract replacement. It is worth noting, however, that introducing aqueous protein extracts to pasta formulations often results in drastic increases in mineral content, likely due to the salts present in the buffer solutions employed for the extraction. Since the most common buffers are prepared from sodium salts, the increases in sodium content in these kinds of formulated foods should be considered.

#### 4.1.2. Gluten-Free Noodles

There are already some studies aiming to produce gluten-free noodles with insects to enhance their nutritional profiles. Musika et al. [76] optimized a riceberry rice flour pasta supplemented with *A. domesticus* powder using a D-optimal mixture design. Riceberry rice is a variety of *Oryza sativa* L. from Thailand. This variety has a distinctive dark purple color and contains phenolic compounds, anthocyanins, and vitamins. For the design, the authors tested several riceberry rice flour to *A. domesticus* flour ratios (in terms of flour substitution, levels ranged from 5.3 to 21.1%) and variable amounts of xanthan gum (from 1% to 5% addition). The design took into consideration different technological, nutritional, biological, and sensory variables. Regarding the nutritional aspects, protein and fat content were assessed. The design rendered a lower limit of 12% and 4.06% and an upper limit of 14.57% and 6.85% for protein and fat, respectively. The optimal formulation, taking into account all the design variables, resulted in that prepared by replacing 14.7% of the riceberry rice flour with *A. domesticus* flour, with a 1.45% addition of xanthan gum.

Finally, Wannasupchue and Wongthahan [77] assessed the effect of different contents of *G. bimaculatus* powder in rice and tapioca noodles formulations. The authors reported incorporating 15 and 30% cricket powder into the formulations, although it is not clearly stated if a replacement or an addition methodology was employed for this. However, results showed drastic increases in all components: crude protein from 6.33% to 16.19 and 23.03% (wb); fat from 1.03% to 2.31 and 5.36% (wb); ashes from 0.20% to 0.68 and 3.37% (wb); and crude fiber from 0.90% to 2.63 and 3.67% (wb) for the control, 15% and 30% replacement, respectively. Although not declared, it can be assumed (by difference) that carbohydrates significantly decreased from 80% to 71 and 58% for the control, 15% and 30% replacement, respectively.

### 4.2. Technology and Quality Evaluation

**Figure 3:**
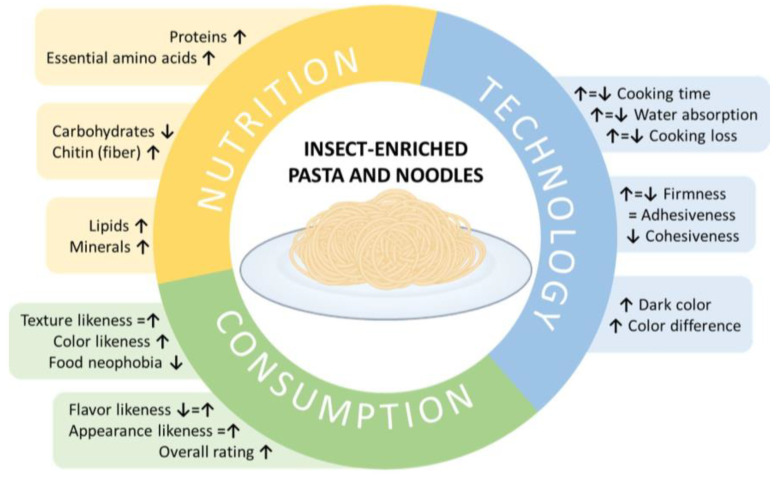
Overall nutritional, technological, and consumption aspects of insect-enriched pasta and noodles. Up and down arrows indicate whether the parameter increased or decreased, respectively, while the equals signs indicate that no significant changes were found. The presence of different signs for the same parameter indicates that independent studies reported different results.

With this in mind, partially replacing semolina, wheat flour, or other flour from cereals, tubers, and roots with insect powders is a promising strategy for producing nutritious and health-promoting pasta and noodles available worldwide. However, beyond the nutritional aspects, there are still other factors that need to be addressed before these kinds of products can be globally spread out. Several authors have tested different kinds of commercially available and laboratory-produced insect flours and extracts in pasta and noodles formulations, evaluating their quality, sensory attributes and consumer attitudes at different levels, alongside their nutritional benefits (Figure 3).

#### 4.2.1. Wheat-Based Products

##### Flour Blends and Premixes

The first step in evaluating the suitability of a flour blend for these types of products is assessing the physical and chemical properties of their primary raw ingredients and, most importantly, how they behave when they are mixed with water or oils. For instance, binary blends of durum wheat semolina and whole *T. molitor* powder were evaluated by Carpentieri et al. [78] at different proportions (95:5; 90:10; 80:20; and 70:30). The authors found that even though the *T. molitor* powder had water and oil absorption capacities 1.5 and 1.8 times higher than semolina, respectively, the blends maintained the same absorption properties as the semolina alone, indicating promising suitability for pasta production. This was probably due to the higher number of soluble components present in the *T. molitor* powder [78] and the emulsifier properties of its proteins [79]. Similar results for *A. domesticus* were found in different studies, with up to 20% [80] and 30% [81] wheat flour substitution. Conversely, other studies reported changes in the water absorption profiles of blends with different ingredients. In these cases, the water absorption capacity was higher in blends prepared with soft wheat flour and *A. domesticus* powder, as the replacement level increased from 25 to 50% [82], or up to 75% [83], and with super wheat flour and *L. migratoria* powder, up to 5% replacement [38]. Given the wide variability in the properties of wheat products and insect derivatives, comparing studies can be challenging. Therefore, it is recommended that the hydration behavior of each blend be evaluated until a more comprehensive database on this matter is established and further studied.

##### Dough Rheology and Microstructure

Understanding the interaction of hydrated polymers in a dough matrix is crucial for developing suitable processes for pasta and noodle production [84,85]. However, as of the moment when this review was written and to the best of the authors’ knowledge, only one study, by Bresciani et al. [80], included empirical rheology (i.e., farinograph, alveograph, extensograph) evaluations of dough in the experimental design. The key findings in this regard were the significant and sometimes progressive changes produced by the use of 5, 10, and 20% of cricket powder (*A. domesticus*) on the dough properties, mostly due to the dilution effect of the gluten proteins. This was reflected in the decreasing farinographic development time and stability, the increasing degree of softening, the decrease of dough extensibility both uni-axial and three-dimensional, and an increase in dough tenacity. No other information on how adding insect-derived products could affect the fundamental rheology of dough, its microstructure, and/or even its empirical behavior was found.

##### Cooking Quality

The analysis of pasta and noodles may be challenging due to the diversity in the type and number of ingredients used in different formulations. However, there is a well-known set of empirical assessments designed to determine their technological properties and quality, i.e., the optimal cooking time (OCT), the water absorption (WA), and the cooking loss (CL, the transference of solids from the pasta or noodles to the water while cooking). This set of evaluations is usually complemented by some others, such as color and texture.

Cricket powder from *A. domesticus* was tested on wheat-based pasta formulations in different studies ranging from 0 to 15% wheat flour replacement [72,86]. When using durum wheat semolina in fettuccine production, Ho et al. [86] found little to no changes in the OCT, WA, and CL of the pasta made with 6.8% semolina replacement with insect flour during cooking compared to a durum wheat semolina pasta with 23.5% whole wheat flour (wheat semolina basis). In fact, Duda et al. [72] stated that *A. domesticus* tagliatelle pasta presented a color resembling commercially available whole wheat flour pasta. However, these authors did find significant and progressive increases in the OCT of durum wheat semolina spaghetti formulated with 5, 10, and 15% *A. domesticus* powder, respectively, and in this case, the CL was progressively decreased with the increase of insect powder concentration, also accompanied by decreasing WA, likely due to the lower amount of starch.

In contrast, when using protein extracts instead of whole insect flours, the results were the opposite. Using water-soluble protein extracts from *A. domesticus* (14%), *T. molitor* (14%), *B. mori* (10 and 15%), or *H. illucens* (15%) on semolina tagliatelle and spaghetti [74,75,87], opposite results were found regarding CL and WA, which significantly increased in most cases, indicating that other components, different from the water-soluble protein fraction of insect meals, could play an important role in this process [74].

On the other hand, when adding other ingredients besides wheat to pasta and noodles formulations, such as egg, the incorporation of *A. domesticus* flour up to 10% also increases the WA and CL significantly [80], while the use of *L. migratoria* and *T. molitor* flours significantly increases the OCT and CL, but decreases the WA when used in a 10% replacement level [73]. Conversely, adding *B. mori* flour up to 10% on buckwheat pasta supplemented with wheat gluten significantly decreased the OCT and strongly increased the WA [88].

Despite the wide variety of formulations tested, it is possible to arrive at some generalities at this point. For instance, wheat pasta can benefit from whole-insect meals in terms of these quality parameters, while the use of water-soluble protein extracts would produce more undesirable changes in this regard. However, when adding other ingredients to the formulation, the effect of the insect meal would depend both on the insect species and the nature of the ingredients used and should be further studied.

##### Color and Texture

Regarding the quality parameters aiming for commercial suitability of pasta and noodles, the color and the texture of the products are critical since consumers would rather choose those products that are more visually appealing. Therefore, the instrumental assessment of these parameters while developing such products is useful for obtaining the desired features.

Several authors have instrumentally evaluated the changes in color on pasta samples through the 1976 CIELAB color space parameters L*, a*, and b*, and the color deviation, ΔE, a mathematically derived parameter that accounts for the color differences between a sample of interest and a reference sample (usually, a control). It is largely established that values of ΔE higher than 3 would be easily noticeable to the naked eye of non-expert consumers [89,90]. In all cases, the parameter L* that is related to white (L* = 100) and black (L* = 0) shades decreased from a whiter value (70.8–78.8) for control pasta to darker values (41.3–64.5) in pasta with different proportions of insect flour or extracts [72,74,75,87]. This led to color differences that would be clearly perceived by the consumers, with ΔE values ranging from 13.2 to 41.3 for both raw and cooked pasta and for several insect species, e.g., *H. illucens* [75], *B. mori* [74,75], *T. molitor* [87], and *A. domesticus* [72,87].

However, when comparing insect-based pasta with a different control, such as one made with whole wheat flour, these changes become less drastic, and it is possible to achieve no perceptible differences for raw pasta [86], which could be more appealing to those consumers interested in healthier foods [72]. Given the above, since insect flours tend to be darker, ranging from pale to dark shades of brown and gray, compared to the common cereal flours employed for pasta production, mixing these flours generally results in darker blends, showing a concentration effect.

On the other hand, the texture of pasta and noodles supplemented with insect powders varies widely depending on the formulation and process. Using whole powders from *A. domesticus* [72] and protein extracts from *A. domesticus* or *T. molitor* [87] up to 14–15% produced slight but significant increases in the pasta firmness when compared to a semolina or wheat flour control, although no changes were found regarding the adhesiveness [87]. However, when comparing against a whole wheat-based control pasta, using 5% cricket powder led to similar results in terms of firmness and springiness, although a strong decrease in cohesiveness was observed [86]. Moreover, adding egg into the formulation produced no changes in firmness when replacing wheat flour with 5% insect meal, and increasing this level to 10% even led to a drastic decrease (ca. 50%) of its firmness [80]. However, in this last study, this behavior could be attributed to the use of the same cooking time (90 s) for all formulations since the authors did not indicate testing the OCT for each individual one.

In contrast, when using *B. mori* powder or protein extracts, results were not exactly conclusive since the few works in this regard measured different textural parameters. Piazza et al. [74] found that using whole *B. mori* powder up to 10% led to a similar cutting force as the control, while this value decreased when using the protein extract. In turn, Hidalgo et al. [75] found that at a 15% level of replacement with *B. mori* or *H. illucens* protein extracts, the pasta firmness did not change. However, in both studies, it was found that the breaking force of the uncooked pasta significantly decreased in these samples.

#### 4.2.2. Non-Wheat-Based Products

If scientific information about wheat-based products elaborated with insects is still scarce, the scenario is even more difficult regarding non-wheat-based products. Only a few studies have addressed this issue, differing greatly in the formulation approach and the set of evaluations performed, strongly limiting the comparison among the studies. However, it is interesting to note that all these works were focused only on two cricket species, i.e., *A. domesticus* [76] and, especially, *G. bimaculatus* [77,91,92,93].

Kiiru et al. [93] tested how adding different proportions of *G. bimaculatus* powder to common gluten-free flours (maize, rice, finger millet, and proso millet) changed the physicochemical properties of the blends (95:5, 90:10, 85:15, 80:20). The authors tested several parameters such as particle size and morphology, color, and hydration properties. Their findings show nutritional improvements, noticeable color changes, and several modifications in the hydration behavior of the different blends, most of them showing a concentration effect. Interestingly, parameters such as water holding capacity, water absorption capacity, and oil absorption capacity were less affected, even though *G. bimaculatus* powder showed higher values than the gluten-free flours, especially regarding water holding capacity.

In noodles, only two studies are suitable for comparison in terms of formulation and insect species, although it would be somewhat limited by the different assessments set. Both studies, by Thongkaew et al. [92] and Wannasupchue and Wongthahan [77], assessed noodles prepared with rice and tapioca flour. The first study added *G. bimaculatus* protein extract powder up to 10%, while the latter added whole *G. bimaculatus* powder up to 30%. In this case, only color and tensile strength can be compared. Both studies found significant and progressive decreases in the L* and b* parameters when the proportion of insect-derived products was increasing. However, when using whole cricket powder, no changes were found for a* [77] in contrast with the decrease found when using the protein extract [92]. Thongkaew et al. [92] found little to no changes in the tensile strength of the samples, up to 8% substitution with insect protein concentrate, followed by a drastic decrease at 10% substitution. When evaluating the same parameter, Wannasupchue and Wongthahan [77] reported significant decreases in both the 15 and 30% replacement formulations in comparison to the control. It is also worth noting that almost no changes were found for any formulation in terms of cooking weight and CL [92].

Musika et al. [76] evaluated the addition of *A. domesticus* powder in a 5–20% range into a riceberry rice and xanthan gum noodle formulation, using a D-optimal mixture design that accounted for color, texture, protein and fat contents, antioxidant properties, and sensory evaluation. The authors found that a mixture of 84.10% riceberry rice flour, 14.45% *A. domesticus* powder, and 1.45% xanthan gum was optimal in terms of product desirability.

## 5. A Way Towards Consumption

### 5.1. Food Neophobia, the Role of Information

In a general scenario, when it comes to introducing unfamiliar foods or ingredients into everyday diets, there are risks of triggering an aversive behavior known as food neophobia, defined as the unwillingness to consume new or unfamiliar foods and the propensity to avoid them [94]. Recently, several scientific studies and reviews have explained how neophobia influences food behavior. In countries that are not culturally familiar with insects as food or ingredients (particularly Western countries), incorporating whole insects into the diets leads to low acceptance of these kinds of products, an outcome mainly driven by neophobia [49,95,96,97].

Conversely, some authors found that introducing insects as powders and extracts (i.e., in a way that they are not seen or associated with the idea of the insect moving or living in the wild) could contribute to reducing food neophobia [1]. Additionally, providing information about nutritional features and benefits of these new ingredients has proved to help with better adoption of insects as food since information can significantly influence consumers’ opinions and attitudes towards food [95,97].

### 5.2. Sensory Analysis, the Role of Development

In the particular case of pasta and noodles meals, consumer attitudes and sensory analysis have demonstrated that using these carriers is an appropriate choice since pasta is generally consumed based on its energy contribution to diet, and in this case, adding a nutritious ingredient such as insects would increase its presence as a convenient food [95]. This is also true for other cereal-based, staple, and savory products [38,98]. Conversely, other products such as sweets, chocolates, and pastries have been shown to be more challenging since consumers tend to eat these kinds of products based only on pleasure [95].

Sensory analysis of pasta and noodles has shown promising results. *A. domesticus* powder proved to be a suitable ingredient for durum wheat (semolina) pasta, with sensory attributes that were scored even higher than those of the control (color, taste, overall rating, texture) by untrained consumers [72]. When compared to a whole-wheat flour pasta served as a dish in a blind study, Ho et al. [97] found that pasta with *A. domesticus* powder was significantly better than the control in terms of overall liking and that there were no significant differences for all the other tested attributes (appearance, aroma, taste, texture, flavor, aftertaste). Moreover, the participants could not tell accurately if the sample contained insects or not. However, when another group of participants was informed about the ingredients in the presented samples, a significantly higher percentage of plate waste was found for those containing insects, which negatively correlated with the taste-liking attribute.

The visual appearance of semolina pasta made with *B. mori* and *H. illucens* powders and protein extracts was reported to be liked by consumers. However, the smell perception of the samples was negative for the powder-made pasta, although it became neutral for the pasta made with the protein extracts [75]. Similar results were found by Çabuk and Yılmaz [73] for erişte egg pasta with *T. molitor* and *L. migratoria*, also reporting notably lower scores mainly for the odor attribute, as well as low values for color and smoothness.

When used in formulations of gluten-free pasta, Biró et al. [88] found that silkworm (*B. mori*) powder helped to mask the buckwheat flavor, which some consumers may dislike, when used at a 10% level. In another study, Wannasupchue and Wongthahan [77] found that *G. bimaculatus* powder yielded excellent sensory performance in rice-tapioca noodles, with scores similar to or higher than the control for all attributes when used at a 15% level. The authors also found that increasing this level to 30% produced a drastic decrease in the scores for all attributes. In fact, Musika et al. [76] found that 14.45% cricket powder (*A. domesticus*) was the optimal formulation by a D-optimal design study of a riceberry rice noodle formulation with 1.45% xanthan gum. In this study, the optimized responses for the formulation were 7.40 for the texture and 7.01 for the overall acceptance.

## 6. Opportunities

**Figure 4:**
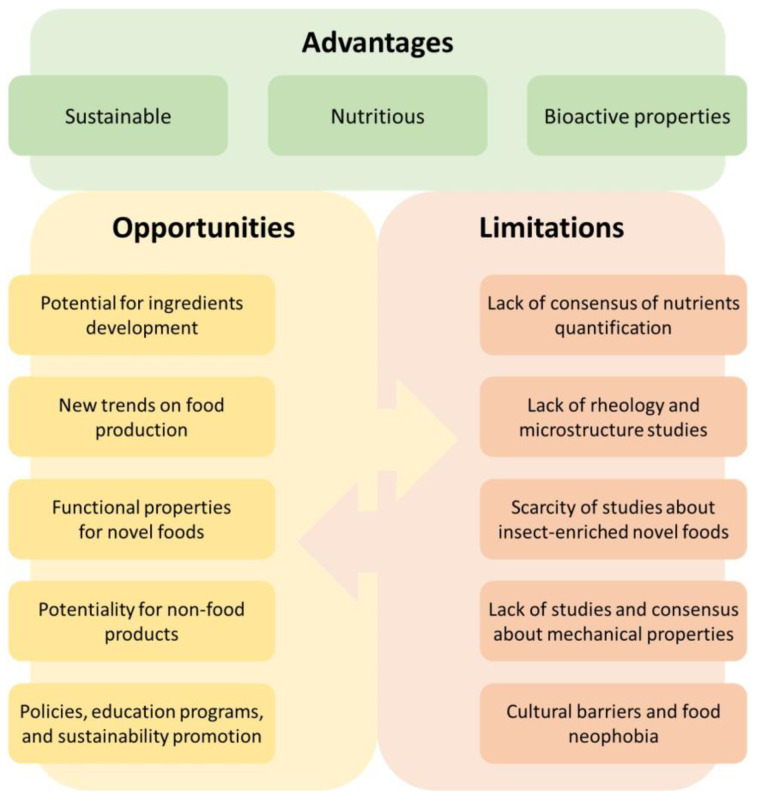
Chart of the key aspects of entomophagy and insect-based novel foods, showing some of the more important relations between their opportunities and current limitations.

Edible insects can play a significant role in the forthcoming ways of production (Figure 4). The wide variety of edible insect species, including those yet to be studied as such, opens the possibility for the development of countless ingredients, derivatives, and food products in general. Moreover, among these new products, new sources of biological activities are yet to be unveiled. The more these commodities are studied, the more they can be understood and then used for human nutrition and health benefits.

Insects are also a source of components with diverse technological functionality. Insect extracts and derivatives can thus profit from the design of food products thanks to their foaming and emulsifying properties. The new trends in food production, such as 3D printing, could benefit from these properties.

Additionally, insects are a significant source of chitin, a nitrogen polysaccharide that can be used as-is or functionalized into chitosan, both with known biological activities (mainly antimicrobial) and functional properties (emulsifier, thickener, stabilizer, binding agent, and so on). Chitinous products, and especially chitosan, have been subjects of interest for the development of new materials (e.g., biocompatible antimicrobial films) for use in food technology, medicine, and even pharmacology and cosmetology [99].

Moreover, since the awareness of how the current food production system contributes to the climate crisis and risks to food security is rising, more and more consumers are turning their habits toward greener and more environmentally friendly alternatives. This could be read as the prelude to a paradigm change in the food production system; thus, researchers, producers, and markets should be attentive to make sure edible insects are included in it. The development of new staple foods, including edible insects, the study of consumer’s responses and attitudes towards these new products, and the adoption of policies and programs to promote their consumption, including all the involved agents (i.e., researchers, educators, policymakers, producers, culinary experts, and so on), are key in this process.

## 7. Limitations

There are still several issues to be addressed in making edible insects a commodity for the food industry (Figure 4). Starting from the basics, the efforts towards accurate protein measurements and nitrogen-to-protein conversion factors in insects, mainly due to the chitin interference, are yet to be studied and systemized. This is a challenging task, especially due to the wide spectrum of edible insect species and even the differences that can be found within the same species in terms of sex, development stage, diets, and more. The huge list of edible species available makes it quite impossible to generalize, and this can be seen as an opportunity (as stated before) and also as a limitation since resources for analyzing such a number of samples are always scarce.

The above-stated for the protein measurement issues is also valid for the proximate composition in general. In order to create a more accurate database for scientists, developers, policymakers, industries, and consumers, the way in which the proximate composition of new ingredients and products is standardized is critical. It is essential to establish an appropriate way to report protein, as well as dietary fiber and carbohydrate values. The standard methods for measuring proteins (the Kjeldahl method) and total dietary fiber (the official methods AOAC 991.43, AOAC 985.29, AACC 32-07.01, and AACC 32-05.01) interfere with each other due to the nitrogen-containing nature of chitin. This scenario leads to critical errors in the estimation of these components, which overlap, and since carbohydrates are generally estimated by difference, the proximate composition tables found elsewhere in the literature are mostly unclear and inaccurate.

In the particular case of pasta products, especially those including insect ingredients, the scarcity of studies addressing dough fundamental rheology, microstructure, and water dynamics makes it difficult to understand how the main components of the dough (gluten proteins, starch, water) interact with the components present in the insect ingredients (insect proteins, chitin). This information is valuable for addressing technological problems, finding new additives, and designing processes. Thus, further studies performed on complex and model systems should be encouraged.

Additionally, several articles have focused on the sensory attributes of pasta products, but there is still a lack of studies addressing mechanical tests performed on uncooked dried pasta and even little consensus among authors on evaluating the textural parameters of cooked pasta. The first group of tests is of utmost importance for handling, packaging, and delivering pasta products, while the latter is critical for predicting their quality on a production line. In both cases, studies should focus on the evaluation of well-established parameters and the accuracy of the reported methods. This is especially important for pasta technology, where the wide variety of formulations, ingredients, and production processes make comparison among studies difficult.

## 8. Conclusions

Ingredients derived from edible insects offer exciting possibilities for the future of human nutrition in a sustainable way. The research and development in this field are still in their early stages, and many opportunities are waiting to be addressed, whether in terms of emerging technologies, product development, or health benefits. Pasta and noodles have already been tested as carriers of insect ingredients by several authors, and they have shown promising results. Nutritionally, different studies demonstrated that pasta and noodles prepared with insect ingredients were higher in protein, fiber, and lipids and lower in carbohydrates. In terms of sensory evaluation, different studies showed that the perception of these novel foods is promising and can benefit from information and familiarity.

Nevertheless, drawing such general conclusions about the technological aspects of these kinds of products is not yet entirely possible. Many factors contribute to this scenario, such as the diversity of ingredients used, processing, methods of assessment, and even the different ways in which the data and results are reported. This issue has become particularly important due to the scarcity of studies on it. However, this situation can be seen not only as a limitation but also as an opportunity since being in these initial stages of the research presents a change for designing new investigations. In this way, further research should aim not only to fill the gaps in knowledge but also to encourage more standardized methods and procedures.

## Figures and Tables

**Table 1:** Advances in biological activities of several insect species intended for food.

Biological Activity	Ingredient	Insect Species	Processing Conditions	Relevant Findings	Study
Antioxidant	Whole flour, hydrolysate powders	*T. molitor*	Flour hydrolyzed with Alcalase or Alcalase and Flavourzyme, as is and after INFOGEST digestion	EC_50_ of DPPH reducing activity was similar for whole flour and hydrolysates Simulated digestion significantly decreased EC_50_ of hydrolysates but not in whole flour	[40]
	Fractioned protein hydrolysates	*A. domesticus*	Flour protein extraction at pH 12 followed by precipitation at pH 4, hydrolyzed with Alcalasa and fractioned with size exclusion and reverse phase chromatography	Size exclusion fractions showed several antioxidant capacities (by DPPH, ABTS, FRAP)	[41]
	Hydro-alcoholic extract	*Coridius chinensis*	Hydro-alcoholic (methanol) extraction of room-temperature dried and grounded specimens	Several antioxidant capacities (SOD, GST, DPPH, ABTS) demonstrated in vitro	[43]
	Decolorated and non-decolorated homogeneous chitosan	*H. illucens*	Chitin from larvae, pupal exuviae, and adults (biomasses) extraction (mineralization, deproteinization, decoloration) followed by homogeneous deacetylation	Free radical scavenging activity (DPPH) of homogeneous chitosan from *H. illucens* was similar (non-decolorated) or higher (decolorated) than other common chitosan sources (shiitake fungi and crab shells)	[44]
Antihypertensive	Hydrolysate powders	*B. mori*	Vacuum microwave oven-dried powder hydrolyzed with Alcalase^®^2.4 L and freeze-dried	LD_50_ ≥ 2000 mg kg^−1^ lowering blood pressure effect in hypertensive rats ≥ 100 mg kg^−1^	[42]
	Fractioned protein hydrolysates	*A. domesticus*	Flour protein extraction at pH 12 followed by precipitation at pH 4, hydrolyzed with Alcalasa and fractioned with size exclusion and reverse phase chromatography	All fractions had ACE inhibition activity	[41]
Anti-inflammatory	Whole flour, hydrolysate powders	*T. molitor*	INFOGEST digested whole flour and flour hydrolyzed with Alcalase or Alcalase and Flavourzyme	Digested samples reduced the expression of several pro-inflammatory genes (TNF-α, IFN-γ, and IL-6) on CACO-2 cells in most cases Some increase of anti-inflammatory cytokine IL-4	[40]
	Hydro-alcoholic extract	*C. chinensis*	Hydro-alcoholic (methanol) extraction of room-temperature dried and grounded specimens	In vitro protein denaturation inhibition: IC_50_ 1592.3 μg mL^−1^ crude extract; identification of known anti-inflammatory compounds (palmitoyl ethanolamide and etodolac glucuronide) in extracts	[43]
Antimicrobial	Decolorated and non-decolorated homogeneous chitosan	*H. illucens*	Chitin from larvae, pupal exuviae, and adults (biomasses) extraction (mineralization, deproteinization, decolorating) followed by homogeneous deacetylation	Minimal inhibitory concentration of 0.15 mg mL^−1^ Higher *E. coli* inhibitory activity than commercial crustaceans’ chitosan at concentrations higher than 0.3 mg mL^−1^	[44]
Antidiabetic	Sericin-derived oligopeptides (SDO) from silk cocoons	*B. mori*	Autoclaving, filtering, and proteolysis of the aqueous extract, followed by centrifugation, ultra membrane filtration, and freeze-drying	In a study with male rats, compared to the diabetic control SDO at 50–200 mg kg^−1^ increased HDL, albumin, and aorta relaxation; decreased blood glucose level, and aorta contraction (at normal control level); recovered pancreas histopathology, and both α and β cells immunohistochemistry features SDO at 100–200 mg kg^−1^ lowered blood triglycerides, alkaline phosphatase; and increased protein body weight gain, and total proteins compared to diabetic control SDO at 200 mg kg^−1^ increased insulin level, decrease uric acid, and reduce 50% eye cataracts compared to diabetic control	[45]
Anti-osteoclastogenic	Insect commercial extract	*G. bimaculatus*	Not reported	Extracts reduced the receptor activator of nuclear factor-κB ligand (RANKL)-dependent osteoclast differentiation, osteoclast maturation traits, and osteoclast bone resorption ability without cytotoxic effect on bone marrow macrophages (up to 5 μg mL^−1^)	[46]

## Data Availability

No new data were created or analyzed in this study. Data sharing is not applicable to this article.
